# Molecular characterization of vancomycin-resistant *Enterococcus faecium* and identification of a clinical strain co-harboring the G2505A mutation associated with linezolid resistance from a tertiary hospital in China

**DOI:** 10.1128/spectrum.00014-26

**Published:** 2026-08-25

**Authors:** Ni Suo, Yujing Wang, Chunguang Chen, Haijun Li, Nan Yi, Xiaogai Li, Cailin Liu, Wanhai Wang

**Affiliations:** 1 Department of Clinical Laboratory, The First Affiliated Hospital of Zhengzhou University 191599, Zhengzhou, China; 2 The First Clinical Medical College of Zhengzhou University 667963, Zhengzhou, China; 3 Department of Clinical Laboratory, The Sixth People’s Hospital of Zhengzhou City, Zhengzhou, China; Purdue University, West Lafayette, Indiana, USA

**Keywords:** vancomycin-resistant *Enterococcus faecium*, *vanHAX*, *vanHMX*, vancomycin, G2505A mutation in the 23S rRNA gene

## Abstract

The rise of vancomycin-resistant *Enterococcus faecium* (VREfm) poses a growing global health threat. This study aimed to characterize the molecular features and resistance mechanisms of clinical VREfm isolates from a Chinese tertiary hospital. Eighteen VREfm isolates were identified using matrix-assisted laser desorption/ionization time-of-flight mass spectrometry (MALDI-TOF MS) and whole-genome sequencing (WGS). Antimicrobial susceptibility testing was performed via the broth microdilution method. Resistance mechanisms and molecular epidemiology were analyzed using WGS and bioinformatics tools. Additionally, plasmid-mediated transfer of vancomycin resistance genes was evaluated through conjugation assays. Among the 18 VREfm isolates, 13 carried only the *vanHAX* gene cluster, while 5 harbored both the *vanHAX* and *vanHMX* gene clusters. Notably, two VREfm strains—EFM250320 and EFM250826—were isolated from the same patient but from specimens of the same type collected at different time points. Strain EFM250320 carried both the *vanHAX* and *vanHMX* gene clusters, whereas strain EFM250826 carried not only the *vanHAX* and *vanHMX* gene clusters, but also a G2505A mutation in the 23S rRNA gene associated with linezolid resistance. Five distinct sequence types (STs) were identified, with ST80 (44.4%, 8/18) being the predominant type.

**IMPORTANCE:**

This study reveals notable drug resistance patterns in vancomycin-resistant *Enterococcus faecium* (VREfm) bacteria at our hospital, with multiple resistance genes identified in the isolates. Notably, one strain was found to concurrently harbor both the *vanHAX* and *vanHMX* gene clusters, along with a G2505A mutation in the 23S rRNA gene that confers resistance to linezolid. The dissemination of the dominant ST80 clone suggests potential intra-hospital transmission. These findings underscore the necessity for enhanced surveillance and stringent infection control measures to curb the spread of such hazardous multidrug-resistant pathogens.

## INTRODUCTION

*Enterococcus faecium* (*E. faecium*) is a commensal bacterium of the gastrointestinal tract while also being most frequently associated with serious bloodstream and urinary tract infections, particularly among immunocompromised and hospitalized patients (1). Enterococci exhibit intrinsic resistance to various classes of antimicrobials (2). In recent decades, the prevalence of acquired antimicrobial resistance (AMR) in *E. faecium* has markedly increased, including the emergence of vancomycin-resistant *Enterococcus faecium* (VREfm) (3).

The VREfm has emerged as a major nosocomial pathogen (4). It presents a severe challenge to global healthcare systems due to its resistance to last-line antimicrobial agents and its association with life-threatening infections (5). Vancomycin resistance in hospital-adapted *E. faecium* can be conferred by several variants of the *van* operon (3). The primary genetic determinants of vancomycin resistance in clinical isolates from Europe, North America, Africa, and Asia are predominantly the *vanA* and *vanB* genes (6, 7). These multidrug-resistant (MDR) pathogens, particularly *vanA*-positive *E. faecium*, can precipitate nosocomial outbreaks that are associated with increased healthcare costs, morbidity, and mortality (8). *VanM* was initially identified in an *E. faecium* isolate from a hospital in Shanghai in 2006 (9). Subsequently, it has become the predominant resistance determinant in vancomycin-resistant *enterococci* (VRE) strains since 2011, exhibiting a higher prevalence compared to *vanA* in the Shanghai region (10). Mechanisms of acquired linezolid resistance have been identified in enterococci. These primarily comprise chromosomal mutations, point mutations in the central loop of domain V of the 23S rRNA, and the acquisition of transferable genes encoding oxazolidinone resistance (8, 11). The inclusion of VREfm on the World Health Organization’s priority pathogen list, coupled with its substantial contribution to the global burden of healthcare-associated infections, underscores the urgent necessity for enhanced surveillance and effective management strategies (12).

We collected a total of 18 *E. faecium* isolates. Among these, 13 VREfm strains carried only the *vanHAX* gene cluster (*vanH* gene, *vanA* gene, and *vanX* gene), while 5 VREfm strains harbored both the *vanHAX* and *vanHMX* gene clusters (*vanH* gene, *vanM* gene, and *vanX* gene). Notably, one VREfm strain was found to carry not only the *vanHAX* and *vanHMX* gene clusters but also a G2505A mutation in the 23S rRNA gene, which mediates linezolid resistance. As the first clinically approved oxazolidinone antibiotic, linezolid serves as a last-resort therapy for severe infections caused by multidrug-resistant Gram-positive pathogens (13). It holds particular value in treating both nosocomial and community-acquired pneumonia, as well as complicated skin and soft tissue infections, including vancomycin-resistant enterococci (14–16).

This study demonstrates that VREfm isolates in a Chinese tertiary hospital setting exhibit a concerning molecular profile. The universal presence of the *vanHAX* gene cluster, its co-occurrence with *vanHMX* in some strains, and the identification of a vancomycin-linezolid co-resistant isolate highlight the evolving complexity of antimicrobial resistance. The predominance of the ST80 clone suggests potential clonal dissemination. These findings emphasize the urgent need for enhanced surveillance and strict infection control measures to contain the spread of such multidrug-resistant, high-risk VREfm strains.

## MATERIALS AND METHODS

### Isolation and identification of bacteria

Bacterial clinical isolates were obtained from The First Affiliated Hospital of Zhengzhou University, a large tertiary teaching hospital in east-central China. All clinical isolates were identified with matrix-assisted laser desorption/ionization time-of-flight mass spectrometry (MALDI-TOF MS) and whole-genome sequencing (WGS). The study included 11 male and 7 female participants, with ages ranging from 41 to 87 years. The VREfm isolates were obtained from various clinical specimens, including urine, blood, bronchoalveolar lavage fluid, drainage fluid, secretion, bile, and pleural fluid. The isolates were primarily obtained from urine (33.3%, 6/18). The isolates EFM230603, EFM241204, EFM250106, EFM250107, EFM250216, EFM250826, and EFM250318 were derived from patients who had received vancomycin therapy prior to the isolation of this vancomycin-resistant isolate. The characteristics of the isolates, together with relevant clinical data, are detailed in Table S1.

### Antibiotic susceptibility profiles

The minimum inhibitory concentrations (MICs) of the VREfm isolates were determined using the broth microdilution method, in accordance with Clinical and Laboratory Standards Institute (CLSI) guidelines (M100-S35) (17). The tested antibiotics included vancomycin, teicoplanin, linezolid, rifampin, nitrofurantoin, tetracycline, levofloxacin, ampicillin, chloramphenicol, ciprofloxacin, erythromycin, daptomycin, and penicillin (Thermo Fisher Scientific). *Enterococcus faecalis* ATCC 29212 (https://www.atcc.org/support/product-technical-support/faqs/citing-atcc-cultures) as the quality control strain.

### Whole-genome sequencing of VREfm isolates

Genomic DNA was extracted via the SDS method (18). The extracted DNA was assessed by agarose gel electrophoresis and quantified with a Qubit 2.0 Fluorometer (Thermo Fisher Scientific). WGS of the isolates was conducted on the short-read Illumina NovaSeq × plus PE150 platform (Novogene Bioinformatics Technology Co., Ltd., Beijing, China), and the reads were assembled using SPAdes v4.0.0 (19). Subsequently, representative strains selected from the isolates were further sequenced using Nanopore long-read sequencing (Novogene Bioinformatics Technology Co., Ltd., Beijing, China), and a hybrid assembly of short and long reads was performed with Unicycler v0.5.1 (20). Genomic sequence similarity among the isolates was analyzed with the CGView Comparison Tool (21). AMRs and chromosomal mutations were identified using ResFinder 4.7.2 (22). The sequence types (STs) were determined via PubMLST (23). 23S rRNA mutations were assessed with LRE-finder 1.0, plasmids detected using PlasmidFinder 2.1, and acquired virulence genes analyzed via VirulenceFinder 2.0 (24).

### Genetic context of resistance genes and phylogenetic relationships

Representative strains were selected for in-depth investigation and subjected to long-read sequencing using Nanopore platforms (25). The genome sequences were annotated with Bakta v1.8.2 (26). Insertion sequence (IS) elements and integrons were identified through the ISfinder database, and annotation results were subsequently verified and refined using NCBI databases (27). Plasmid genome sequences were comparatively analyzed with Proksee (28). The genetic contexts of antibiotic resistance genes were visualized using ChiPlot (29). Complete genome sequences of nine *E. faecium* strains were downloaded from the NCBI database and combined with all whole-genome sequences of *E. faecium* strains isolated in this study. All genomes (*n* = 27) were annotated using Prokka v1.14.6, followed by multiple sequence alignment with Roary v3.12.0. A maximum-likelihood phylogenetic tree was then constructed using RAxML v8.2.12 (30) and visualized with TVBOT (31).

### Bacterial conjugation assays

To investigate the transferability of vancomycin resistance gene-carrying plasmids, filter-mating conjugation assays (32) were performed using *Enterococcus faecalis* JH2-2 as the recipient strain (33). Putative transconjugants were selected on brain heart infusion agar plates supplemented with 16 µg/mL vancomycin + 32 µg/mL fusidic acid. Subsequently, transconjugants were confirmed using MALDI-TOF MS and WGS.

## RESULTS

### Isolation and molecular characterization of bacterial strains

**Fig 1:**
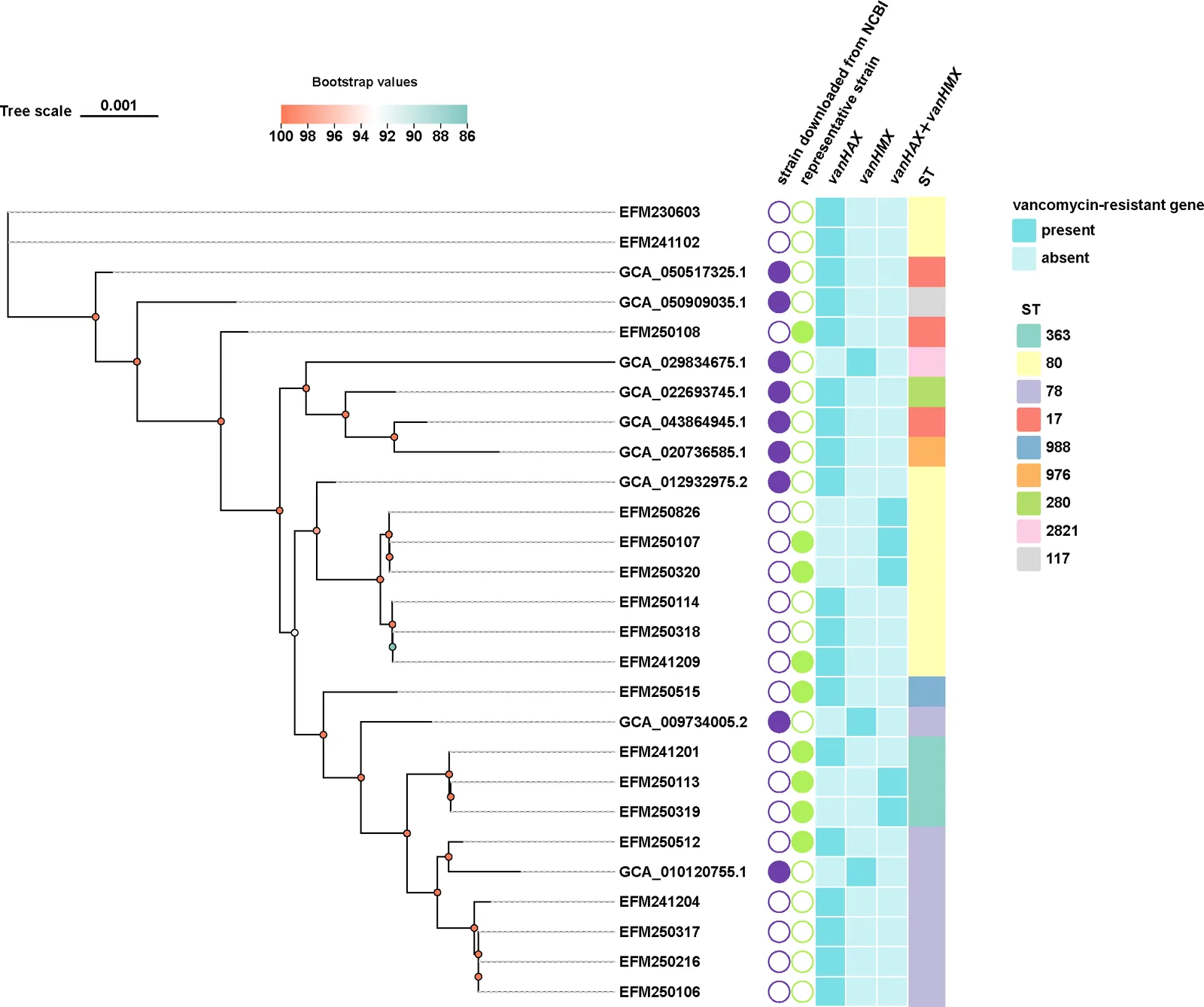
The phylogenetic tree was constructed with TVBOT software using nine *Enterococcus faecium* strains from public NCBI data and 18 vancomycin-resistant *Enterococcus faecium* clinical isolates.

A total of 18 vancomycin-resistant *E. faecium* strains (MIC ≥ 256 µg/mL) were isolated between June 2023 and August 2025. All VREfm isolates showed variable susceptibility to other tested antibiotics. All VREfm isolates were resistant to rifampin (100%, 18/18), levofloxacin (100%, 18/18), ampicillin (100%, 18/18), ciprofloxacin (100%, 18/18), and penicillin (100%, 18/18) (Table 1). Complete antibiotic susceptibility profiles are provided in Table S2. Among all 18 isolates, 13 carried only the *vanHAX* gene cluster, 4 carried both the *vanHAX* and *vanHMX* gene clusters, and one isolate harbored the *vanHAX* and *vanHMX* gene clusters along with a G2505A mutation in the 23S rRNA gene that mediates linezolid resistance (Table 2). All isolates were positive for the virulence genes *acm* and *efaAfm*. Sequence types were identified among the 18 isolates: ST80 (*n* = 8), ST78 (*n* = 5), ST363 (*n* = 3), ST988 (*n* = 1), and ST17 (*n* = 1). All strains were assigned to clonal complex 17 (CC17) (Table S1). Phylogenetic analysis of the 27 VREfm isolates resolved them into three distinct clusters. This analysis further indicated that strains sharing the ST were genetically closely related (Fig. 1).

**TABLE 1:** Antimicrobial susceptibility profiles of VREfm isolates^*b*^

Antibiotics	VREfm isolates (*n* = 18)			
	*S*%	*I*%	*R*%	SDD %
Teicoplanin	1 (5.6%)		17 (94.4%)	
Linezolid	17 (94.4%)		1 (5.6%)	
Rifampin			18 (100%)	
Nitrofurantoin		5 (27.8%)	13 (72.2%)	
Tetracycline	1 (5.6%)		17 (94.4%)	
Levofloxacin			18 (100%)	
Ampicillin			18 (100%)	
Chloramphenicol	7 (38.9%)	10 (55.5%)	1 (5.6%)	
Ciprofloxacin			18 (100%)	
Erythromycin	1 (5.6%)		17 (94.4%)	
Daptomycin			11 (61.1%)	7^*a*^ (38.9%)
Penicillin			18 (100%)	

^*a*^ According to the CLSI M100-S35 guidelines regarding daptomycin susceptibility, the SDD is specifically applicable to serious infections caused by *E. faecium*. It is recommended to consult an infectious diseases specialist for further guidance.

^*b*^ VREfm, vancomycin-resistant *Enterococcus faecium*.

**TABLE 2:** Characteristics and vancomycin resistance mechanisms of VREfm isolates^*a*^

Isolate	Species	ST (clonal complex)	AMRs	Virulence genes	23S rRNA mutations
EFM241201	*E. faecium*	363 (CC17)	*vanHAX*	*acm*, *efaAfm*	–^*b*^
EFM241102	*E. faecium*	80 (CC17)	*vanHAX*	*acm*, *efaAfm*	–
EFM230603	*E. faecium*	80 (CC17)	*vanHAX*	*acm*, *efaAfm*	–
EFM241204	*E. faecium*	78 (CC17)	*vanHAX*	*acm*, *efaAfm*, *espfm*, *hylEfm*	–
EFM250106	*E. faecium*	78 (CC17)	*vanHAX*	*acm*, *efaAfm*, *espfm*, *hylEfm*	–
EFM250107	*E. faecium*	80 (CC17)	*vanHAX*, *vanHMX*	*acm*, *efaAfm*	–
EFM250108	*E. faecium*	17 (CC17)	*vanHAX*	*acm*, *efaAfm*, *espfm*	–
EFM241209	*E. faecium*	80 (CC17)	*vanHAX*	*acm*, *efaAfm*, *espfm*	–
EFM250512	*E. faecium*	78 (CC17)	*vanHAX*	*acm*, *efaAfm*, *espfm*	–
EFM250113	*E. faecium*	363 (CC17)	*vanHAX*, *vanHMX*	*acm*, *efaAfm*	–
EFM250114	*E. faecium*	80 (CC17)	*vanHAX*	*acm*, *efaAfm*	–
EFM250515	*E. faecium*	988 (CC17)	*vanHAX*	*acm*, *efaAfm*, *espfm*	–
EFM250216	*E. faecium*	78 (CC17)	*vanHAX*	*acm*, *efaAfm*, *hylEfm*	–
EFM250317	*E. faecium*	78 (CC17)	*vanHAX*	*acm*, *efaAfm*, *hylEfm*	–
EFM250318	*E. faecium*	80 (CC17)	*vanHAX*	*acm*, *efaAfm*	–
EFM250319	*E. faecium*	363 (CC17)	*vanHAX*, *vanHMX*	*acm*, *efaAfm*	–
EFM250320	*E. faecium*	80 (CC17)	*vanHAX*, *vanHMX*	*acm*, *efaAfm*, *espfm*	–
EFM250826	*E. faecium*	80 (CC17)	*vanHAX*, *vanHMX*	*acm*, *efaAfm*, *espfm*	G2505A

^*a*^ ST, sequence type; AMRs, acquired antimicrobial resistance genes; CC17, clonal complex 17; *E. faecium*, *Enterococcus faecium*; VREfm, vancomycin-resistant *Enterococcus faecium.*

^*b*^ –, no 23S rRNA mutations were detected.

### Genetic context analysis of *vanHAX* and *vanHMX* genes

**Fig 2:**
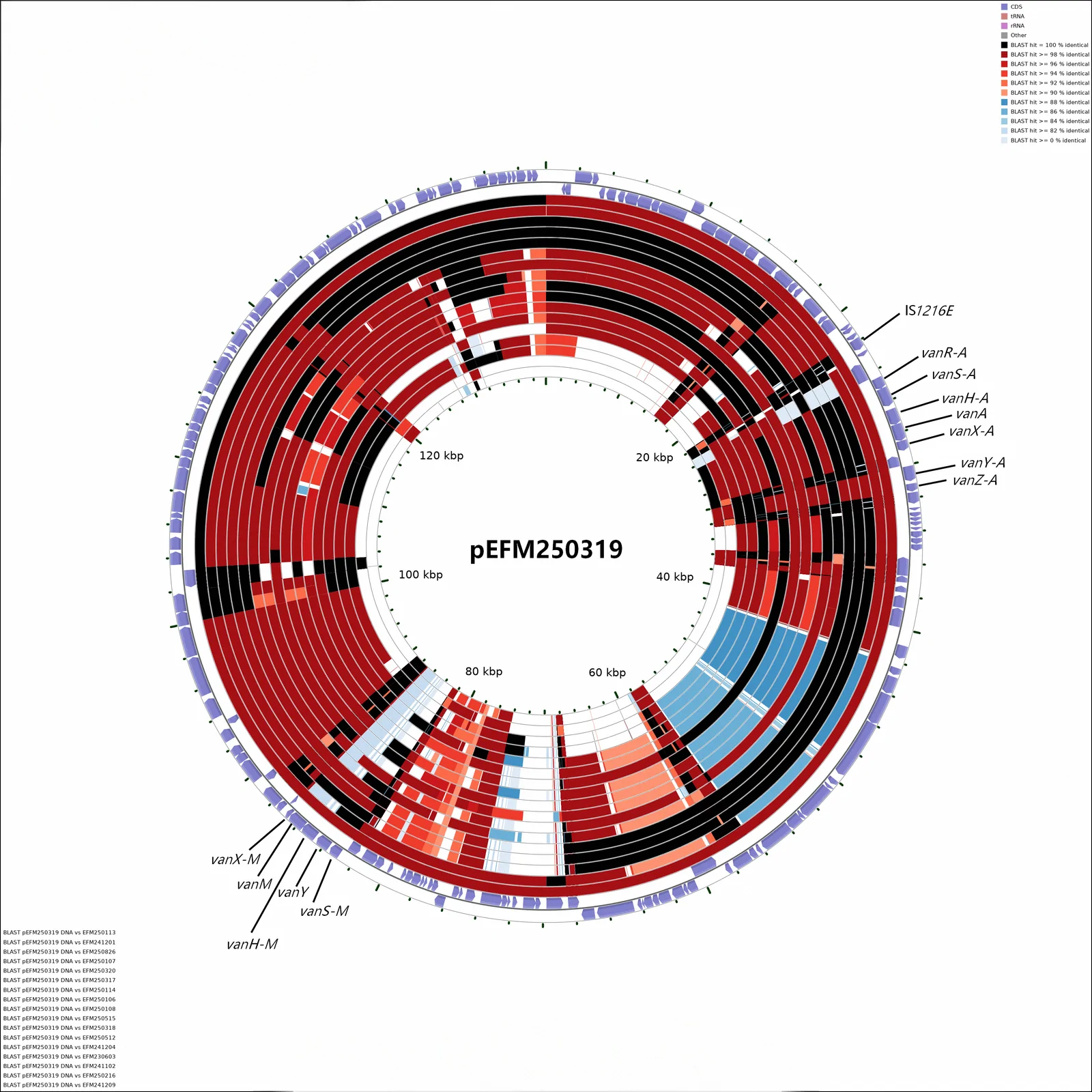
Using the CGView Comparison Tool, we analyzed the genome sequence similarity among VREfm strains. The sequential order from the outer to the inner circle in the circular diagram corresponds to the strain order annotated in the lower-left corner.

**Fig 3:**
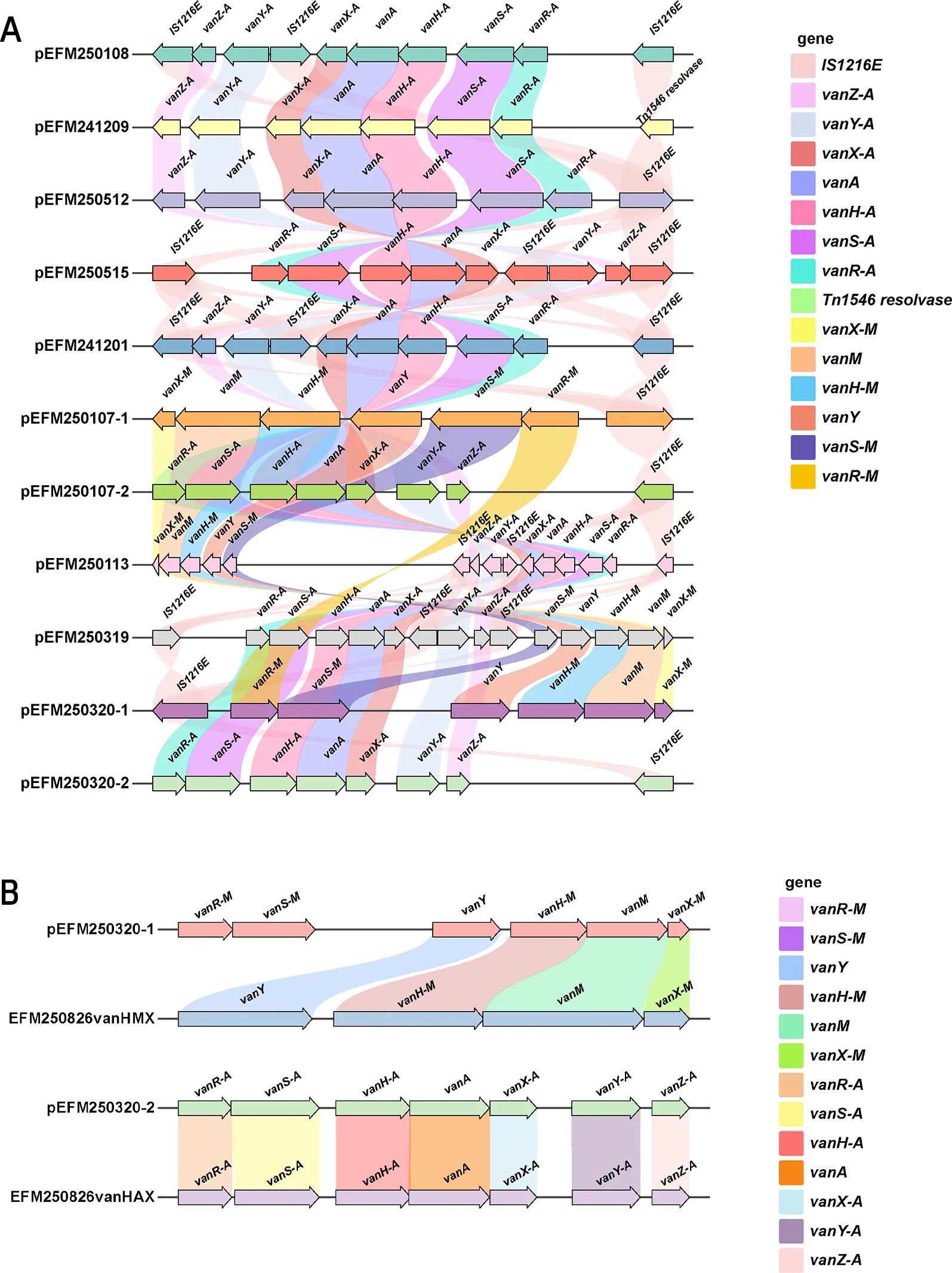
Genetic context analysis of the *vanHAX* gene cluster (**A**) and *vanHMX* gene cluster (**A and B**) was performed using ChiPlot. Arrow directions indicate the orientation of the genes and mobile genetic elements.

**Fig 4:**
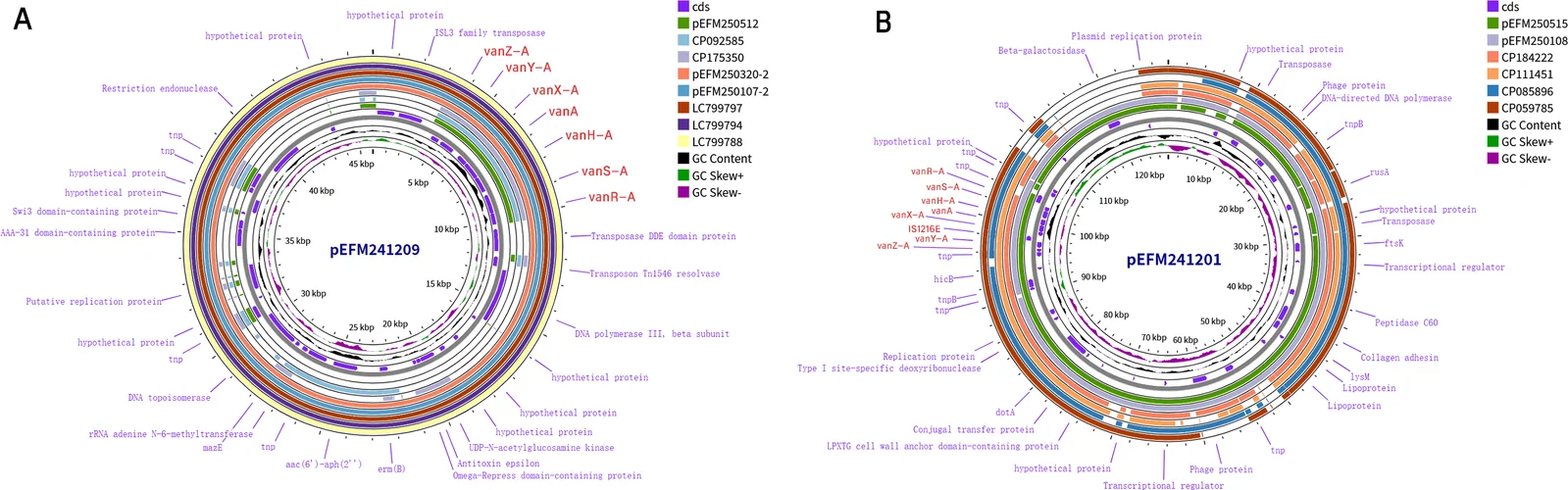
The circular genome maps in panel **A** (plasmids pEFM241209, pEFM250512, pEFM250320-2, pEFM250107-2, and the reference strain) and panel** B** (plasmids pEFM241201, pEFM250515, pEFM250108, and the reference strain) were generated using Proksee software.

**Fig 5:**
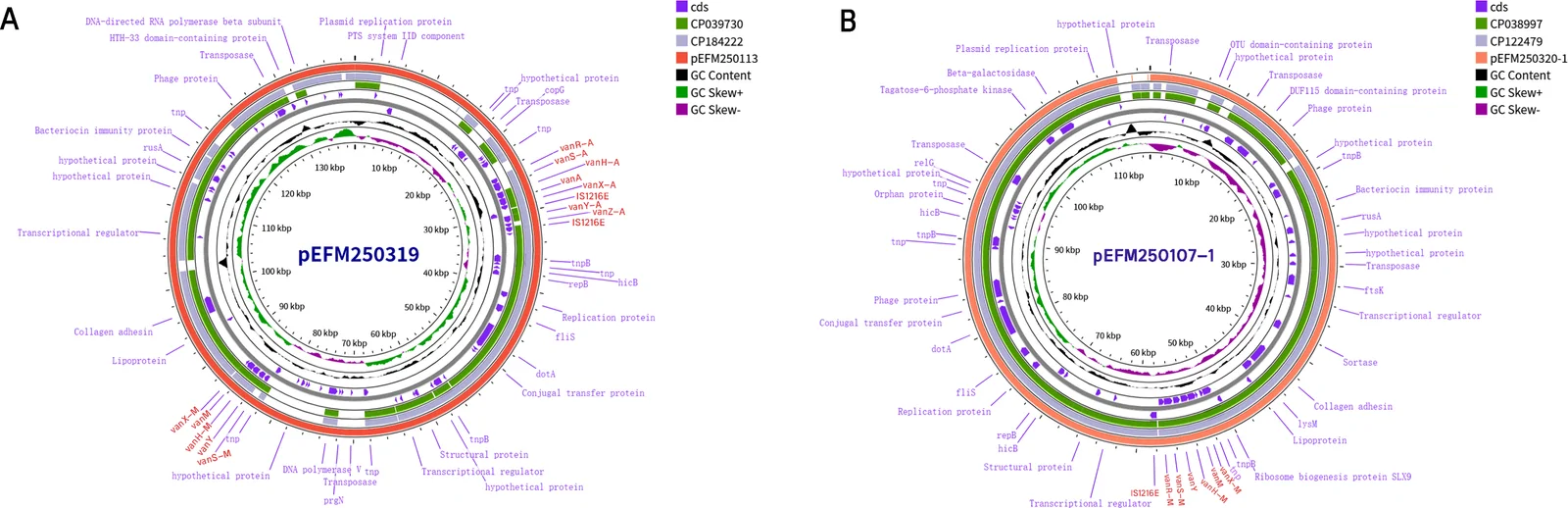
The circular genome maps presented in panel **A** (plasmids pEFM250319 and pEFM250113, along with the reference strain) and panel **B** (plasmids pEFM250107-1 and pEFM250320-1, along with the reference strain) were generated using Proksee software.

The genomic context surrounding the *vanHAX* gene cluster consistently formed the *vanRSHAXYZ* structure. However, the structural clusters flanking the *vanHMX* gene cluster showed incomplete similarity among different strains, with variations across the isolates (Fig. 2). Nine representative VREfm strains—EFM250108, EFM241209, EFM250512, EFM250515, EFM241201, EFM250107, EFM250113, EFM250319, and EFM250320—were selected from the 18 isolates for further analysis. Both plasmids pEFM250113 and pEFM250319 belong to the RepA_N family with the rep17 replication module, while pEFM250512 belongs to the Inc18 family with the rep2 replication module. No replication genes were detected in pEFM250108, pEFM241209, pEFM250515, pEFM241201, pEFM250107-1, pEFM250107-2, pEFM250320-1, and pEFM250320-2 using the PlasmidFinder tool (34). BLASTN analysis demonstrated high similarity between the vancomycin resistance gene-carrying plasmids from the nine representative VREfm strains and the reference plasmids, with the query coverage and nucleotide identity data provided in Table S3. The genetic environment of the *vanHAX* gene cluster can be categorized into two types. Type I*_vanHAX_* comprises the arrangement *vanR-vanS-vanH-vanA-vanX-vanY-vanZ* (Fig. 3A), which is found in pEFM241209, pEFM250512, pEFM250107-2, and pEFM250320-2 (Fig. 4A). Type II*_vanHAX_* consists of *vanR-vanS-vanH-vanA-vanX*-IS*1216E-vanY-vanZ* (Fig. 3A) and is present in pEFM241201, pEFM250515, pEFM250108 (Fig. 4B), pEFM250319, and pEFM250113 (Fig. 5A). Similarly, the surrounding genetic context of the *vanHMX* gene cluster can be divided into two types. Type I*_vanHMX_*, represented by pEFM250319 and pEFM250113 (Fig. 5A), has the structure *vanS-vanY-vanH-vanM-vanX* (Fig. 3A). Type II*_vanHMX_*, which is characterized by the gene arrangement *vanR-vanS-vanY-vanH-vanM-vanX* (Fig. 3A), includes pEFM250107-1 and pEFM250320-1 (Fig. 5B).

### Characterization of VREfm strain with co-resistance to vancomycin and linezolid

Two VREfm isolates, designated EFM250320 and EFM250826, were sequentially isolated from bile samples of the same patient—the former collected from the Cardiology Ward on 5 March 2025, and the latter from the Department of Infectious Diseases on 20 August 2025. Phylogenetic analysis revealed that EFM250320 and EFM250826, together with strain EFM250107 (isolated from a bronchoalveolar lavage fluid sample of another patient in the Intensive Care Unit on 28 January 2025), formed a well-supported monophyletic clade. All strains within this clade were identified as belonging to the ST80 (CC17) and carried the *vanHAX* and *vanHMX* vancomycin resistance gene clusters (Fig. 1). Notably, EFM250320 and EFM250107 clustered directly and closely, while EFM250826 was positioned peripherally within the clade and harbored a unique G2505A mutation in the 23S rRNA gene, which confers linezolid resistance. Prior to the isolation of strain EFM250320, the patient had been treated with cefoperazone-sulbactam. Before the isolation of strain EFM250826, the patient had received meropenem, cefoperazone-sulbactam, vancomycin, and linezolid. The genomic segment EFM250826vanHAX, harboring the *vanHAX* gene cluster from strain EFM250826, exhibited the gene order *vanR-vanS-vanH-vanA-vanX-vanY-vanZ*, which was identical to that observed in pEFM250320-2. In contrast, the genomic segment EFM250826vanHMX, also containing a *vanHMX* gene cluster from strain EFM250826, lacked detectable *vanR* and *vanS* genes when compared to pEFM250320-1 (Fig. 3B).

### Bacterial conjugation assays

Five strains (EFM250108, EFM250515, EFM241201, EFM250319, and EFM250318) successfully underwent conjugative transfer of the *vanHAX* gene cluster. In addition, strain EFM250113 achieved successful transfer of both the *vanHAX* and *vanHMX* gene clusters. The conjugation frequency for the *vanHAX* cluster ranged from 1.6 × 10^−8^ to 7 × 10^−6^, while that for the *vanHMX* cluster was 3.2 × 10^−7^ (Table S4).

## DISCUSSION

The VREfm poses a significant public health threat, frequently causing opportunistic invasive infections that exhibit broad resistance to available antibiotic therapies (3). Linezolid-resistant and vancomycin-resistant *E. faecium* has become a growing concern in hospitals, as it can cause large-scale outbreaks and develop resistance to antibiotics (8, 35, 36).

All 18 VREfm isolates exhibited high-level vancomycin resistance and belonged to clonal complex 17 (CC17), with sequence type ST80 being the most prevalent (44.4%, 8/18). This finding differs from the results reported in previous studies (37–39), in which ST17 was identified as the predominant ST type among VREfm isolates in a hospital in Sichuan, China (37), and ST78 was reported as the dominant clone in both a Chinese hospital (38) and a hospital in Taiwan (39), while it aligns with those described in other studies (40–42), all of which demonstrated that ST80 was the most prevalent ST type among VREfm isolates.

Recent studies have aimed to establish connections between phenotypic and genotypic drug resistance, as well as between bacterial species and resistance genes, in *Enterococcus* (43–45). For example, the *vanA* genotype is characterized by acquired high-level resistance to both vancomycin and teicoplanin and is predominantly detected in VREfm (45). In contrast, *vanM*-positive VRE exhibits heterogeneous resistance to both vancomycin and teicoplanin (45). In our study, all isolates carried the *vanHAX* gene cluster, while five isolates (EFM250107, EFM250113, EFM250319, EFM250320, and EFM250826) also harbored the *vanHMX* gene cluster. All isolates exhibited high-level resistance to vancomycin. However, their susceptibility to teicoplanin varied: one isolate (EFM241204) had a teicoplanin MIC of 256 µg/mL, eight isolates showed MICs of 128 µg/mL, another eight had MICs of 64 µg/mL, and one isolate (EFM250512) displayed an MIC of 8 µg/mL. Despite carrying the *vanHAX* gene cluster, isolate EFM250512 exhibited a teicoplanin MIC of 8 µg/mL, a finding that is not entirely consistent with the above-reported phenotypic patterns associated with this genotype.

The *vanA* operon is typically located on Tn*1546*-like transposons and confers high-level resistance to both vancomycin and teicoplanin (46). The MDR region carrying the *vanHAX* gene cluster in all strains had formed a Tn*1546*-type transposon (Tn*1546vanRSHAXYZ*). The plasmids pEFM250108, pEFM250515, pEFM241201, pEFM250113, and pEFM250319 all contain an IS*1216E* insertion sequence located between the *vanX* gene and the *vanY* gene, with its orientation opposite to that of the *vanRSHAXYZ* operon. In the four VREfm strains carrying the *vanHMX* gene cluster, the MDR region formed a van*SYHMX* structure in two strains (50%, 2/4), while the remaining strain exhibited a van*RSYHMX* gene cluster configuration, which is consistent with previous studies (47). The genetic context surrounding the *vanHMX* gene cluster varies among the different plasmids that carry it.

We isolated VREfm strains EFM250320 and EFM250826 from bile specimens of the same patient, collected from the Cardiology Ward on 5 March 2025, and from the Department of Infectious Diseases on 20 August 2025, respectively. Both EFM250320 and EFM250826 belong to ST80 (CC17) and carry the *vanHAX* and *vanHMX* gene clusters, indicating a clonal infection or colonization. The critical finding is the emergence of linezolid resistance in the later isolate, EFM250826, mediated by the G2505A mutation in the 23S rRNA gene. This observation differs from previous reports on the co-resistance mechanisms to vancomycin and linezolid (8, 48, 49). Our study suggests an evolutionary pathway for the development of multidrug resistance in a hospital-adapted clone.

Phylogenetic reconstruction provided crucial context for this evolutionary event. EFM250320 and EFM250826, together with an ST80 (CC17) VREfm strain (EFM250107) isolated from an intensive care unit patient, formed a well-supported monophyletic clade, suggesting the potential circulation of a common nosocomial lineage within the healthcare facility. The close clustering of EFM250320 with EFM250107 indicates a high degree of relatedness between these strains, possibly reflecting a recent common ancestor or a direct transmission link. In contrast, the peripheral positioning of EFM250826 within the same clade strongly supports its descent from a lineage closely related to EFM250320, with the observed genetic divergence possibly accumulating during the within-host evolutionary process. This divergence is notably marked by the acquisition of the G2505A mutation in the 23S rRNA gene.

The antimicrobial treatment history of the patient may provide plausible selective pressure driving this microevolution. Prior to the isolation of the VREfm strain EFM250320, the patient received cefoperazone-sulbactam. However, before the isolation of the strain EFM250826, the treatment regimen included meropenem, cefoperazone-sulbactam, vancomycin, and linezolid. Exposure to linezolid likely established a strong selective environment that favored the emergence and fixation of the G2505A mutation in the 23S rRNA gene in the pre-existing VREfm population.

Detailed analysis of the vancomycin resistance gene clusters revealed further genomic diversity. While the *vanHAX* gene cluster in EFM250826 (designated EFM250826vanHAX) maintained an intact gene order (*vanRSHAXYZ*) identical to that found on plasmid pEFM250320-2, the *vanHMX* cluster (EFM250826vanHMX) exhibited a notable structural difference: the absence of detectable *vanR* and *vanS* genes compared to the plasmid-borne cluster pEFM250320-1. This finding suggests potential genomic reorganization or partial deletion within this locus, underscoring the dynamic nature of resistance determinants even within a closely related clonal background.

In summary, this study of 18 VREfm isolates underscores the predominance of the high-risk CC17 lineage and ST80. While all isolates harbored the *vanHAX* cluster and exhibited high-level vancomycin resistance, teicoplanin MICs varied—including one susceptible isolate—suggesting phenotypic deviation from the classical *vanA* pattern. Furthermore, the study suggests a concerning evolutionary trajectory of a high-risk VREfm clone within a hospital setting. We observed the progression of an ST80 (CC17) clone from vancomycin resistance to dual vancomycin-linezolid resistance via acquisition of the G2505A mutation in the 23S rRNA gene. Additionally, structural variations in the *vanHAX* and *vanHMX* gene clusters highlight the genomic diversity of resistance determinants. Our findings emphasize the urgent necessity for enhanced infection control measures and genomic surveillance to curb the dissemination of such multidrug-resistant pathogens in healthcare environments.

## Data Availability

The sequence data generated in this study have been submitted to the NCBI BioProject database (https://www.ncbi.nlm.nih.gov/bioproject/) under accession number PRJNA1397320.

## SUPPLEMENTAL MATERIAL

Table S1 spectrum.00014-26-s0001.xlsx Characteristics and clinical data of VREfm isolates.

Table S2 spectrum.00014-26-s0002.xlsx Profiles of antibiotic susceptibility in VREfm strains.

Table S3 spectrum.00014-26-s0003.xlsx Plasmids and their highly similar reference strains.

Table S4 spectrum.00014-26-s0004.xlsx Characteristics and conjugation frequencies of VREfm strains used in conjugation assays.

Table S5 spectrum.00014-26-s0005.docx Quality assessment of genomic DNA from vancomycin-resistant *E. faecium* isolates for whole-genome sequencing.

ASM does not own the copyrights to Supplemental Material that may be linked to, or accessed through, an article. The authors have granted ASM a non-exclusive, world-wide license to publish the Supplemental Material files. Please contact the corresponding author directly for reuse.
