# Supplementary material for: Molecular characterization of vancomycin-resistant Enterococcus faecium and identification of a clinical strain co-harboring the G2505A mutation associated with linezolid resistance from a tertiary hospital in China
Source: Microbiol Spectr. 2026 Aug 25;14(10):e00014-26. doi: 10.1128/spectrum.00014-26 (PMC13689489; doi:10.1128/spectrum.00014-26)
Supplement: Supplementary file 1 [file spectrum.00014-26-s0005.docx]

Supplementary Table 5: Quality assessment of genomic DNA from vancomycin-resistant *E. faecium* isolates for whole-genome sequencing

Fig. A and Fig. B: Numbers 1-4 represent strains EFM250320, EFM250113, EFA250319, and EFM250107, respectively.

Fig. C and Fig. D: Number 2 corresponds to strain EFM250108, while numbers 5-8 correspond to strains EFM241209, EFM241201, EFA250512, and EFM250515, respectively.

(1) Fig. A and Fig. D

Pulsed field agarose gel electrophoresis

Lane M: 48Kb GeneRuler High Range DNA Ladder

Sample loading was performed according to the following protocol:

Concentration 0–20 ng/μL: load 5 μL of undiluted sample.

Concentration 20–30 ng/μL: load 4 μL of undiluted sample.

Concentration 30–60 ng/μL: load 2 μL of undiluted sample.

Concentration 60–90 ng/μL: load 1 μL of undiluted sample.

Concentration 90–150 ng/μL: load 2 μL of a 3-fold diluted sample.

Concentration 150–250 ng/μL: load 2 μL of a 5-fold diluted sample.

Concentration 250–500 ng/μL: load 2 μL of a 10-fold diluted sample.

Concentration 500–1000 ng/μL: load 2 μL of a 20-fold diluted sample.

(2) Fig. B and Fig. C

Agarose gel electrophoresis

In the figure, lane M-1 contains the Trans 15K Plus DNA ladder with a loading volume of 2 µL, and lane S contains the standard sample loaded at 5 µL (10 ng/µL). The sample loading protocol was as follows:

Concentration 0–10 ng/µL: load 5 µL of undiluted sample.

Concentration 10–20 ng/µL: load 3 µL of undiluted sample.

Concentration 20–30 ng/µL: load 2 µL of undiluted sample.

Concentration 30–60 ng/µL: load 1 µL of undiluted sample.

Concentration 60–90 ng/µL: load 0.5 µL of undiluted sample.

Concentration 90–150 ng/µL: load 1 µL of a 3-fold diluted sample.

Concentration 150–250 ng/µL: load 1 µL of a 5-fold diluted sample.

Concentration 250–500 ng/µL: load 1 µL of a 10-fold diluted sample.

Concentration 500–1000 ng/µL: load 1 µL of a 20-fold diluted sample.
